# The neurobiology of motivational anhedonia in patients with depression

**DOI:** 10.1007/s11682-025-00999-7

**Published:** 2025-03-31

**Authors:** Sigrid Breit, Niklaus Denier, Nicolas Mertse, Sebastian Walther, Leila M. Soravia, Andrea Federspiel, Roland Wiest, Tobias Bracht

**Affiliations:** 1 University Hospital of Psychiatry and Psychotherapy, University of Bern, Bern, Switzerland; 2 Translational Imaging Center (TIC), Swiss Institute for Translational and Entrepreneurial Medicine, University of Bern, Bern, Switzerland; 3 Department of Psychiatry, Psychosomatics, and Psychotherapy, Center of Mental Health, University Hospital of Würzburg, Würzburg, Germany; 4 Institute of Diagnostic and Interventional Neuroradiology, University of Bern, Bern, Switzerland

**Keywords:** Anhedonia, Depression, Diffusion tensor imaging, Resting state functional MRI

## Abstract

Anhedonia is a core feature of depression. It contains a consummatory and a motivational aspect. Whilst much neuroimaging research in patients with depression focused on the consummatory aspect of anhedonia, less is known about its motivational aspect. This study aimed to explore the neurobiology of networks related to motivational anhedonia. Thirty-eight patients with major depressive disorder (MDD) and 19 healthy controls underwent diffusion-weighted and resting state functional magnetic resonance imaging (rs-fMRI). For assessment of motivational anhedonia, we summed the values of the CORE non-interactiveness score, and the items 1 (hopelessness) and 7 (work and activities) of the Hamilton Depression Rating Scale. Whole-brain voxel-wise statistical analysis of fractional anisotropy (FA) data was performed using Tract-Based Spatial Statistics (TBSS). Additionally, we performed a whole-brain comparison of integrated local correlation of rs-fMRI signal (LCOR), to investigate regional functional differences between patients and healthy controls. Whole brain correlations between motivational anhedonia and measures of structural and functional connectivity (FA, and LCOR) were calculated. TBSS-analyses revealed reduced FA in the left superior longitudinal fasciculus (SLF) in patients with MDD. LCOR was reduced in patients with depression in an adjacent cluster localized in bilateral precunei. Within patients, there was a positive correlation between motivational anhedonia and LCOR in the precunei and a negative correlation in bilateral sensorimotor areas. FA-values did not show significant correlations. These findings suggest that motivational anhedonia in depression is linked to alterations of functional connectivity within bilateral precunei. Observed white matter microstructural alterations in the SLF do not show such an association.

## Introduction

Anhedonia, the loss of pleasure in previously enjoyed activities, is a core feature of depression (Der-Avakian & Markou, 2012; Pizzagalli, 2022; Rizvi et al., 2016). According to the Diagnostic and Statistical Manual of Mental Disorders (DSM-5) it is one of two main criteria for diagnosing a depressive episode (APA, 2013). The DSM-5 extends the historical definition of anhedonia by focusing on diminished interest in addition to diminished pleasure in previously enjoyed activities. This conceptualization contains both a motivational (interest) and a consummatory (pleasure) aspect of reward. Even though the motivational aspect of anhedonia is related to core behavioral and clinical features of depression, (e.g. loss of interest in previously enjoyed activities) research focusing on anhedonia mainly investigated its consummatory characteristics (Bracht et al., 2015b; Der-Avakian & Markou, 2012; Pizzagalli, 2022; Treadway, 2016).

Anhedonia is of central clinical relevance because it is predictive for both antidepressive outcome and time to remission (Khazanov et al., 2020; McMakin et al., 2012; Uher et al., 2008, 2012). It is also a core feature of melancholic depression; a subtype characterized by severe anhedonia, psychomotor retardation and marked neurobiological alterations (Bracht et al., 2014; Denier et al., 2020; Mertse et al., 2022). Furthermore, melancholic depression differs from non-melancholic depression regarding response to distinct treatment modalities (e.g. pharmacotherapy, psychotherapy, electroconvulsive therapy) (Brown, 2007; Szmulewicz et al., 2024; Undurraga et al., 2020; van Diermen et al., 2019). Interestingly, the motivational aspect of anhedonia seems to possess higher specificity than the consummatory aspect for distinguishing patients with depression from psychiatric patients with other diagnoses, such as negative symptoms in schizophrenia (Kaiser et al., 2017; McGlinchey et al., 2006). Furthermore, motivating patients to engage in desired activities (i.e. behavioral activation) is one of the core features of behavioral therapy in depression with proven efficacy (Cuijpers et al., 2007). Thus, impairments of motivation and drive are important features that are relevant for diagnosing and treating depression. However, it is more difficult to capture and to assess motivational in contrast to consummatory anhedonia (Pizzagalli, 2022; Treadway & Zald, 2011).

The reward system is of central importance to mediate both motivational and consummatory aspects of anhedonia in depression (Borsini et al., 2020; Bracht, Linden et al., 2015b). Motivational aspects of anhedonia are associated with wanting, a process that has been extensively investigated with a focus on the dopaminergic reward system (Szczypinski & Gola, 2018). There is broad evidence from translational animal studies and imaging studies in humans to suggest that (dopaminergic) projections from the ventral tegmental area (VTA) to the nucleus accumbens (NAcc) and to the orbitofrontal cortex (OFC) mediate wanting and consecutively reward seeking behavior (Berridge et al., 2009). The most replicated finding in functional magnetic resonance imaging (MRI) studies investigating wanting (e.g., reward anticipation or mechanisms initiating approach behavior) in patients with depression is a blunted activation in the ventral striatum (for literature review see (Borsini et al., 2020). However, motivational processes extend wanting and are influenced by stimulus-reward associations that lead to interest/ desire, anticipation and effort to attain a reward, followed by a hedonic response, which in turn forms novel associations between a stimulus and its perceived reward (Pizzagalli, 2022; Rizvi et al., 2016). Therefore, networks beyond the dopaminergic reward system that are important for action planning and goal directed behavior might also be involved in motivational aspects of anhedonia in depression.

It is the aim of this study to investigate the neuronal underpinnings of motivational anhedonia in major depressive disorder (MDD) (Der-Avakian & Markou, 2012; Pizzagalli, 2022; Treadway & Salamone, 2022). Given that we assume a pathology on a network level, we investigate white matter microstructure of neuronal pathways connecting spatially distributed brain regions using Tract-Based Spatial Statistics (TBSS) (Smith et al., 2006). Alterations of white matter microstructure have been repeatedly reported in patients with depression suggesting a network pathology (Al-Sharif et al., 2024; Bracht, Linden et al., 2015b). This includes findings of associations between white matter microstructure, consummatory anhedonia (Bracht et al., 2014; Bracht, Mertse, Bracht et al., 2022a, b) and deficits of drive (reduced activity levels) (Bracht et al., 2012, 2018; Walther et al., 2012; Wuthrich et al., 2024). In addition, we explore resting state functional MRI (rs-fMRI) alterations of the brain. To supplement structural connectivity analyses of white matter, we explore rs-fMRI using integrated local correlation (LCOR), a local activity measurement defined as the integration of the spatial correlation function of each voxel (Deshpande et al., 2009). Like regional homogeneity (ReHo) it is a measurement of local low-frequency fluctuations. Interestingly, local activity such as ReHo may be related to metabolic activity in animal models (Sun et al., 2021). However, in comparison to ReHo, LCOR shows better performance in discriminating different tissue types, is less prone to physiological noise and less dependent on voxel resolution (Deshpande et al., 2009). Both methods (TBSS and LCOR) focus on local alterations and therefore complement each other aiming at identifying local alterations in brain structure and function. We hypothesized reduced fractional anisotropy (FA) and reductions of local functional connectivity in patients with MDD in regions of the reward system and in extended networks that are crucial for motivation and action planning (Treadway & Salamone, 2022; Walther et al., 2019; Wuthrich et al., 2024). We assumed that these change in white matter microstructure and local functional connectivity are associated with motivational anhedonia.

## Methods

### Participants

Participants were recruited at the University Hospital of Psychiatry and Psychotherapy and have been included in previous studies (Bracht et al., 2022a, 2024; Bracht, Mertse, Bracht et al., 2022a, b, 2023; Denier et al., 2024; Denier, Grieder, Denier et al., 2023, 2024a, b; Mertse et al., 2022). Inclusion criteria were a diagnosis of MDD according to the Diagnostic and Statistical Manual of Mental Disorders (DSM-5) by the American Psychiatric Association (APA, 2013), age between 18 and 65 years, right-handedness and completed assessments of the Hamilton Depression Rating Scale (Hamilton, 1967) and the CORE (Parker & Hadzi-Pavlovic, 1996). Exclusion criteria were neurological disorders, psychiatric comorbidities as assessed with the Mini International Neuropsychiatric Interview (MINI) (Sheehan et al., 1998), personality disorders which were screened using the Structured Clinical Interview for DSM-IV Axis II (SCID-II) (Wittchen et al., 1997) and contraindications to perform an MRI-scan. This resulted in 38 patients with MDD. Diagnosis was made by the treating psychiatrist and confirmed using the MINI (Sheehan et al., 1998). Depression severity was assessed with the 21-item depression rating scale (HAMD) (Hamilton, 1967) and the 21-item self-report Beck Depression Inventory (BDI-II) (Beck et al., 1996). Psychomotor retardation was assessed with the CORE and handedness with the Edinburgh Handedness Inventory (Oldfield, 1971). Out of the 38 patients 32 took antidepressive medication (selective serotonin reuptake inhibitors (SSRI): *n* = 7; serotonin noradrenaline reuptake inhibitors (SNRI): *n* = 14; bupropion: *n* = 4; tricyclic antidepressants: *n* = 8; tranylcypromine: *n* = 1). In 17 patients augmentation strategies were applied (lithium: *n* = 13, atypical antipsychotics: *n* = 4). Furthermore, two patients took zolpidem for sleep induction.

### Assessment of motivational anhedonia

Motivational anhedonia is defined as “diminished motivation to pursue” a hedonic response (Treadway & Zald, 2011). Core aspects of motivation include emotional reactivity/ interactiveness (building a stimulus-reward association and generating interest), expectancies about the future (generating reward anticipation), and the computation of effort/ building an action plan (Kring & Barch, 2014; Rizvi et al., 2016; Treadway & Salamone, 2022; Treadway & Zald, 2011). For a more detailed description we refer to (Rizvi et al., 2016). These features are captured by the CORE sub-score for non-interactiveness (CORE-NI), the HAMD-item 1 (hopelessness), and the HAMD item 7 (work and interest). Based on this concept, we calculated an approximation for motivational anhedonia. We summed adjusted variables the CORE sub-score for non-interactiveness (CORE-NI), the HAMD-item 1 (hopelessness), and of the HAMD-item 7 (work and interest). Given that numeric ranges of the scales vary (HAMD items 0–4, CORE non-interactiveness 0–18) and a least common multiple (lcm) of $\:lcm\left(\text{4,18}\right)=36$, HAMD-items were multiplied by 9, and the CORE non-interactiveness was multiplied by 2 to achieve equal weighting of the three items: *Motivational anhedonia = 9 х HAMD*_(item 1)_ + 9 х HAMD_(item 7)_ + 2 х CORE_NI_). This resulted in a score of motivational anhedonia that ranged from 0 (no motivational anhedonia) to a maximum of 108 (severe motivational anhedonia).

Nineteen healthy right-handed controls were recruited. Inclusion criteria were the absence of any present or past psychiatric disorder as assessed with the MINI and the SCID-II screening questionnaires. Exclusion criteria were identical to the patient group. Healthy controls underwent the same assessments as the patient group. All participants provided written informed consent and received reimbursement for participation. The study was approved by the local cantonal ethics committee (KEK-number: 2017 − 00731).

### MRI data acquisition

Structural and functional MRI data were acquired with a 3 Tesla Magnetom Prisma scanner (Siemens, Erlangen, Germany) and a 64-channel head and neck coil at the University Hospital of Bern. For acquisition of high-contrast T1-weighted images, we used a bias-field corrected MP2RAGE sequence with two gradient echo images (INV1 and INV2) and a T1-weighted image (UNI). Parameters of the MP2RAGE sequence were: 256 Slices, FOV = 256 × 256, 256 × 256 matrix, 1 × 1 × 1 mm3 isotropic resolution, TR = 5000 ms, TE = 2.98 ms, TI = 700 ms and T2 = 2500 ms. Diffusion weighted images (DWI) with 64 non-collinear directions were acquired using a spin-echo echo-planar sequence. DWI parameters were: 64 × b = 1000 s/mm2, 1 x b = 0 s/mm2, 60 Slices, FOV = 269 × 269, 128 × 128 matrix, 2.2 × 2.2 × 2.2 mm3 isotropic resolution, TR = 6200 ms, TE = 69 ms. A continuous resting-state fMRI scan with condition eyes closed was acquired by echo planar imaging (EPI) with the following parameters: 300 volumes with 60 slices per volume, FOV = 230 × 230, 104 × 104 matrix, 2.2 × 2.2 × 2.2 mm3 isotropic resolution, TR = 1300 ms, TE = 37 ms.

### Data analyses

#### Tract-based Spatial statistics (TBSS)

Voxel-wise statistical analysis of FA data was performed using FSL TBSS software (Smith et al., 2006). FA data were projected onto a mean FA tract skeleton, before applying voxel-wise cross-subjects statistics. The tract skeleton was thinned using an FA threshold > 0.2. Group comparisons between patients and healthy controls, as well as exploratory correlational analysis with motivational anhedonia and FA of the fibre skeleton were then performed using permutation tests with *n* = 5000, corrected for multiple comparisons and threshold-free cluster-enhancement (TFCE) with *p* < 0.05. We extracted FA values of significant clusters of group comparisons for intermodal correlations applying a level of significance of *p* < 0.05.

#### Integrated local correlation (LCOR)

We analyzed rs-fMRI using the CONN 20b toolbox (Whitfield-Gabrieli & Nieto-Castanon, 2012). Pre-processing steps include realignment and B0 field map correction of EPI volumes, co-registration to structural T1 MP2RAGE volumes, segmentation and normalization of T1 volumes (and co-registered EPI volumes) to MNI space of structural and functional volumes and smoothing (FWHM kernel of 8 × 8 × 8 mm) of normalized EPI volumes. We applied band-pass filtering (0.008–0.09 Hz) to remove physiological signals and regress nuisance variables of each time series within segmented white matter and cerebrospinal fluid signal and of 12 realignment parameters. Scrubbing of outlier volumes with global BOLD signal or framewise displacement (FD) higher than the 97th percentile was performed using the Artefact Detection Tools (ART) toolbox implemented in CONN. Percentage of excluded volumes did not significantly differ between groups (patients 3.2 ± 3.8% vs. HC 3.1 ± 3.1%, *p* = 0.90). Additionally, for every subject, we computed mean FD of motion parameters and mean DVARS, which is the spatial root mean square of the BOLD signal after temporal differencing. In this sample we did not have to exclude subjects (exclusion criteria: mean FD or mean DVARS higher than two standard deviations above the mean). There was a significant group difference in mean FD (patients 0.162 ± 0.06 vs. HC 0.129 ± 0.052, *p* = 0.04) but none in mean DVARS (patients 0.170 ± 0.075 vs. HC 0.158 ± 0.048, *p* = 0.515). Rs-fMRI time-series were used to compute local correlation (LCOR) maps. LCOR maps measure the local coherence at each voxel, reflecting the strength and direction (positive and negative) of short-range connectivity between a given voxel and its neighboring areas weighted by a Gaussian convolution (25 mm FWHM) (Deshpande et al., 2009). Short-range connections were computed from the matrix of bivariate correlation coefficients between the BOLD time series from each pair of voxels, estimated using a singular value decomposition of the z-score normalized BOLD signal with 64 components separately for each subject. Last, LCOR measures across voxels were rank sorted and normalized separately for each individual subject using a Gaussian inverse cumulative distribution function with zero mean and unit variance. Whole brain voxel-wise comparison of LCOR maps between patients and healthy controls, were both performed with general linear models using mean FD as regressors. We performed analysis with a voxel threshold of *p* < 0.001 and with a False Discovery Rate (FDR) correction of *p* < 0.05. We performed exploratory whole brain correlational analyses of regional LCOR and motivational anhedonia within patients and applied a voxel threshold of *p* < 0.05 and an FDR of *p* < 0.05. Furthermore, we extracted LCOR values of significant clusters of group comparisons for intermodal correlations.

### Intermodal correlational analyses

We performed exploratory Spearman r correlations between clusters of significant group differences of FA and LCOR for all subjects. To investigate group differences (patients and HC) between the intermodal correlations, we proceeded as follows using Python scripting. First, we converted the Spearman r correlation coefficients into z values using Fisher’s t-z transformation: $\:z=0.5\text{ln}\frac{1+r}{1-r}$. Then we calculated the standard error (SE) of the difference between the two z-values, where $\:{n}_{patients}$ and $\:{n}_{HC}$ are the sample sizes: $\:SE=\sqrt{1/{n}_{patients}-3/{n}_{HC}-3}$. Finally, the difference between z-scores were divided with the SE ($\:{z}_{diff}\:=\frac{{z}_{patients}-{z}_{HC}}{SE}$) transformed into p-values using the cumulative distribution function of NumPy.

## Results

Age and sex did not differ between patients with depression and healthy controls. Six patients had double-depression since many years (range 8 to 27 years). For detailed information see Table 1.

**Table 1:** Group differences in demographics and clinical characteristics

	Patients (*n* = 38)	Controls (*n* = 19)	Statistics
Age (years)	44.6 ± 12.2	42.1 ± 13.4	*p* = 0.50
Sex (female/ male)	20/18	8/11	*p* = 0.56
Duration of episode (months)	12.1 ± 10.1	N/A	N/A
Number of episodes	3.0 ± 2.1	N/A	N/A
HAMD-total	22.2 ± 5.5	0.7 ± 1	*p* < 0.001 ***
BDI-total	27.9 ± 9.2	1.6 ± 2.4	*p* < 0.001 ***
CORE-total	12.5 ± 7.5	0.2 ± 0.8	*p* < 0.001 ***
Motivational anhedonia	55.1 ± 15.5	0 ± 0	*p* < 0.001 ***

***: highly significant

### Results of TBSS analyses

**Fig. 1:**
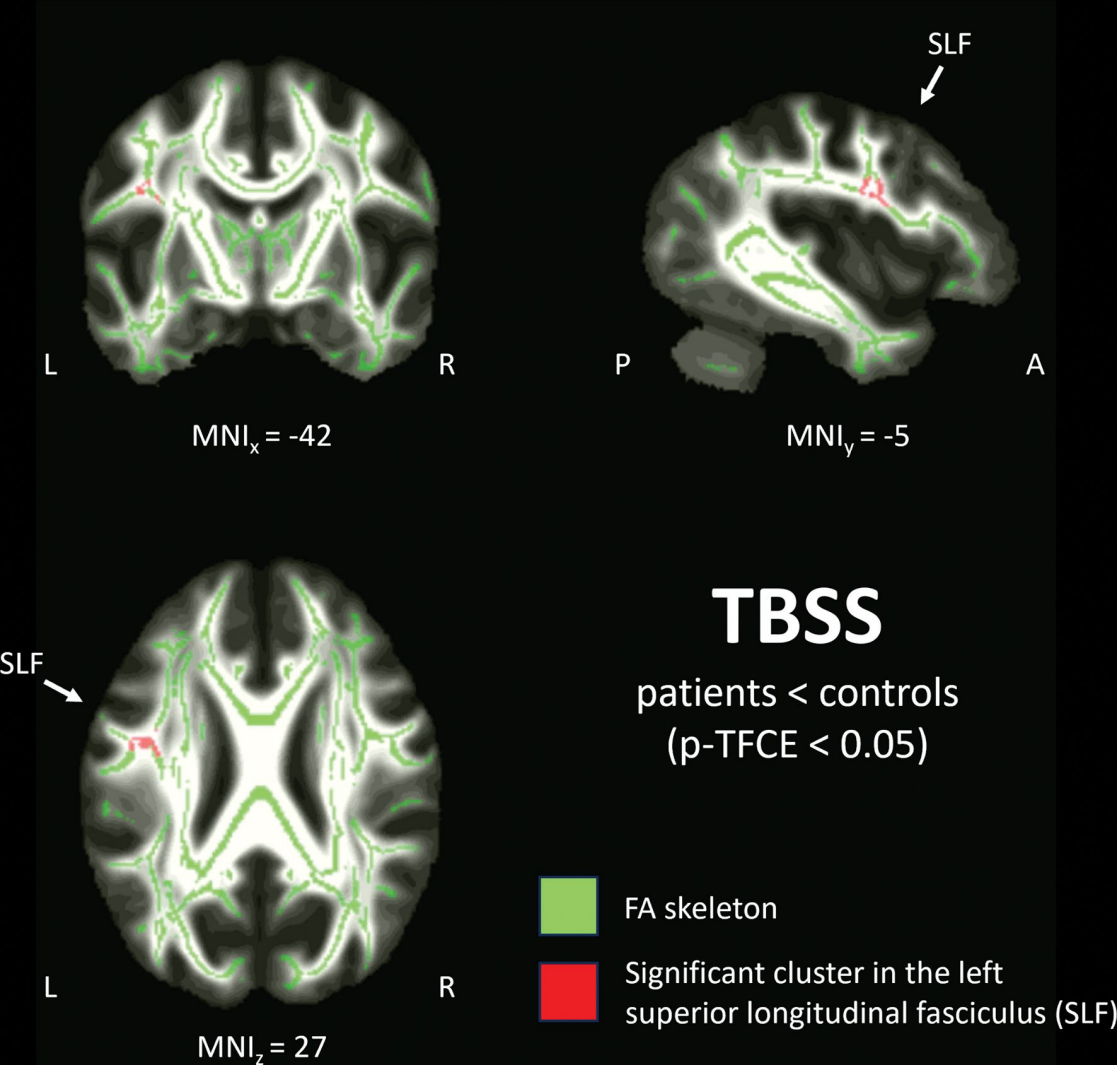
Significant FA TBSS cluster in the superior longitudinal fasciculus (SLF)

There were significant group differences (reduced FA in MDD) in multiple regions, including the left superior longitudinal fasciculus (SLF) and superior, anterior and posterior corona radiata (see Table 2; Fig. 1). There were no significant results for contrast patients > healthy controls. Whole-brain correlation within the TBSS skeleton revealed no significant positive or negative correlation of FA values with motivational anhedonia within patients with MDD.

**Table 2:** Significant FA group differences of TBSS analysis (patients < healthy controls)

White matter tract	Hemisphere	Voxels	*p*-TFCE	MNI X	MNI Y	MNI Z
Superior longitudinal fasciculus	LH	402	0.04	-42	-5	27
Superior corona radiata	RH	86	0.047	17	10	31
Anterior corona radiata	LH	81	0.046	-19	44	16
Anterior corona radiata	LH	56	0.046	-28	32	10
Posterior corona radiata	LH	51	0.047	-18	-37	33

LH: left hemisphere; MNI: Montreal neurological institute atlas; RH: right hemisphere

### Results of LCOR analyses-state functional MRI analyses

**Fig. 2:**
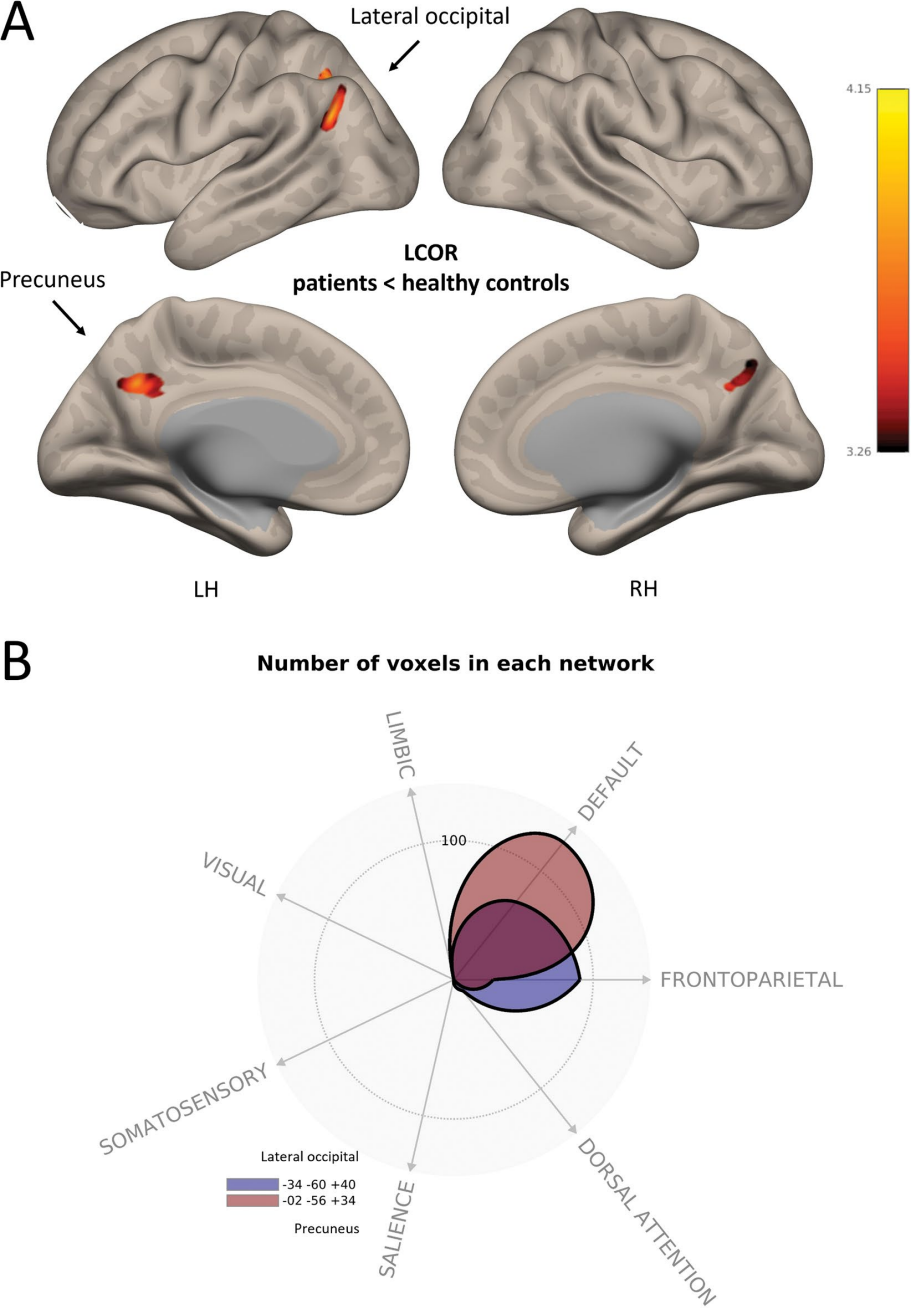
Reduced LCOR in bilateral precuneus in patients with MDD **A**: Significant lower LCOR in patients with MDD. **B**: Overlap of significant clusters and canonical brain networks

**Fig. 3:**
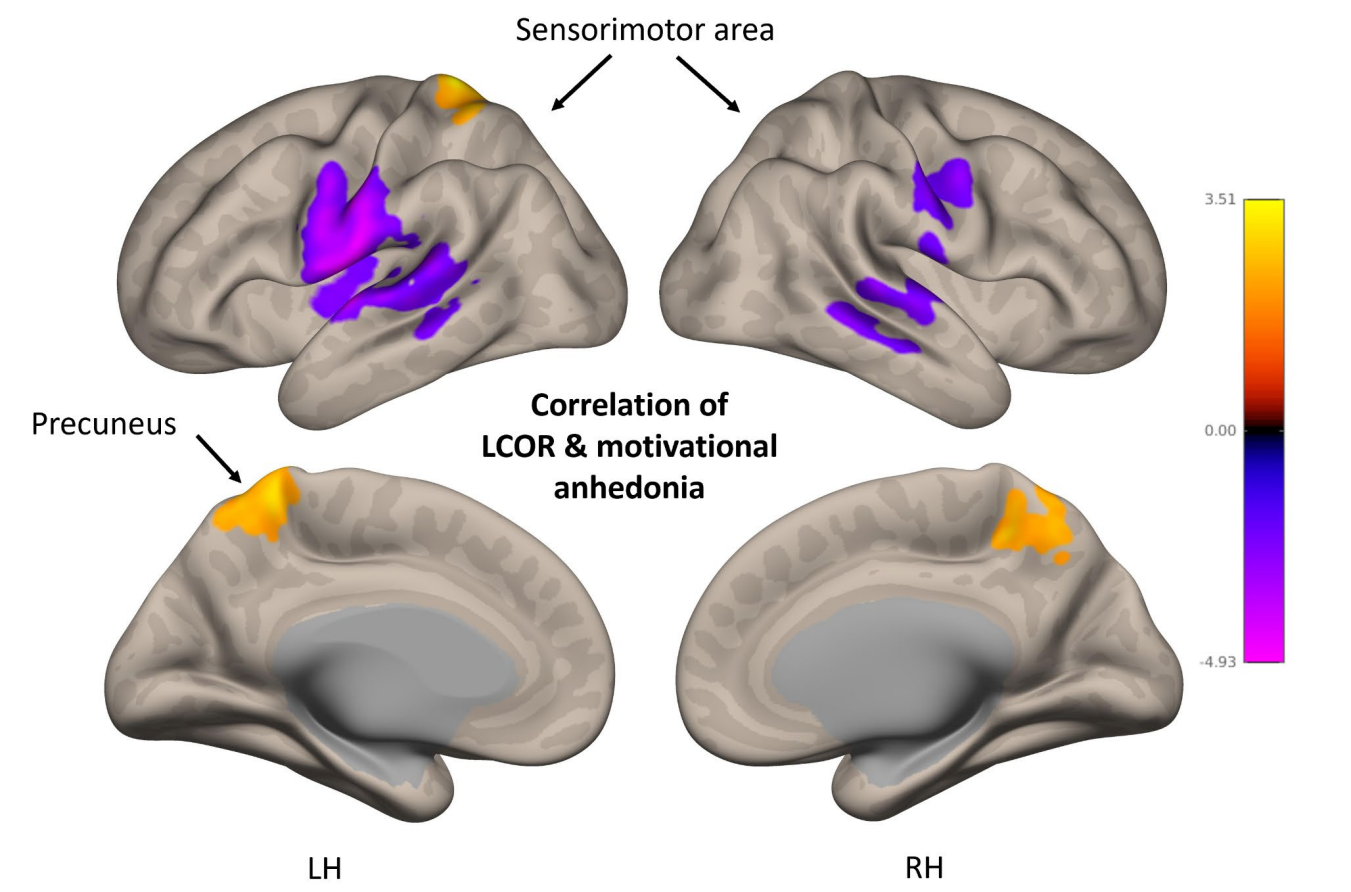
Positive and negative association of LCOR and motivational anhedonia in patients

Whole-brain group comparison revealed significantly lower LCOR-values within patients with depression in comparison to healthy controls (see Table 3; Fig. 2). There were no significant results for contrast patients > healthy controls. Whole-brain correlations within patients revealed a significant positive association of LCOR-values and motivational anhedonia within bilateral precuneus and a negative association in the bilateral sensorimotor area (see Table 4; Fig. 3).

**Table 3:** Significant group differences of LCOR analysis (patients < healthy controls)

Region	Hemisphere	Voxels	*p*-FDR	MNI X	MNI Y	MNI Z
Lateral occipital cortex	LH	253	0.004	-34	-60	40
Precuneus	BH	204	0.006	-2	-56	34

BH: both hemispheres; LH: left hemisphere; MNI: Montreal neurological institute atlas

**Table 4:** Positive and negative associations of LCOR and motivational anhedonia within patients

Association with motivational anhedonia	Direction	Voxels	*p*-FDR	MNI X	MNI Y	MNI Z
Left sensorimotor area	positive	3124	< 0.001	-48	-10	12
Bilateral precuneus	negative	1117	0.045	-22	-44	74
Right sensorimotor area	negative	1075	0.045	66	-32	02

BH: both hemispheres; LH: left hemisphere; MNI: Montreal neurological institute atlas

### Intermodal correlations

Within all subjects, there was a trend association between LCOR in the precuneus and FA in the left SLF (*r* = 0.22, *p* = 0.099). There were no significant correlations between FA in the left SLF and LCOR in the left occipital cortex (*r* = 0.092, *p* = 0.494). There were no significant group differences in intermodal correlations (FA in the left SLF and LCOR in precuneus: *p* = 0.57; FA in the left SLF and LCOR in the left occipital cortex: *p* = 0.61).

## Discussion

It was the aim of this study to investigate the neurobiological underpinnings of motivational anhedonia in patients with MDD. We investigated local alterations of FA (using TBSS) and LCOR, a measure investigating resting state functional connectivity strength between a voxel and adjacent areas. Within patients we found reduced LCOR values in bilateral precunei and a positive association of LCOR and motivational anhedonia in bilateral precunei. Furthermore, we found a negative association of motivational anhedonia and LCOR within bilateral sensorimotor areas in patients. In addition, we identified reduced FA values in patients with depression in various clusters, the largest cluster was localized in the left SLF. However, we found no associations between FA values and motivational anhedonia.

The precuneus and the SLF are both integral components of the brain’s network involved in complex cognitive functions. The precuneus is a core hub of the default mode network (DMN) and plays an important role for self-referential processes, visuo-spatial imagery, and retrieval of episodic memories (Cavanna & Trimble, 2006). In addition, the precuneus forms part of a “paracingulate network” which forms part of larger executive network (Dadario & Sughrue, 2023). Amongst other functions, this paracingulate network has been linked to initial response to reward, reward-anticipation and effort, and planning and performance monitoring (Cao et al., 2019; Dadario & Sughrue, 2023; Oldham et al., 2018). The SLF connects the parietal lobe (including precuneus) with the frontal lobe (including prefrontal and premotor gyri) (Catani et al., 2002) and facilitates language processing (mapping sound to articulation), the integration of sensory information, executive function, and motor planning (Janelle et al., 2022; Saur et al., 2008; Vergani et al., 2021). We found reduced LCOR-values in patients with depression in bilateral precunei suggesting a local dysfunction. In addition, we there was a positive correlation between LCOR and motivational anhedonia. Positive correlations between rs-fMRI connectivity measures and anhedonia have been described before e.g. (Bracht et al., 2022b; Morris et al., 2019). The finding is also in line with observations of a functional hyperactivation in animal models with depression (Cao et al., 2010; Friedman et al., 2016). Therefore, it is possible that the positive association between LCOR and motivational anhedonia in bilateral precunei represents a (dysfunctional) compensatory mechanism in patients with depression with motivational anhedonia. Another explanation may be that patients with pronounced motivational anhedonia show a more pronounced failure to down-regulate DMN activity in (Sheline et al., 2009), which may also be associated with increased rumination (Hamilton et al., 2015). Finally, there may be an impact of antidepressive medication on DMN rs-fMRI-measures (Cui et al., 2021).

Our results suggest a functional disturbance in a region that is in close proximity to the SLF, a pathway that is crucial for integrating information of distinct modalities and for goal directed behaviour (Janelle et al., 2022; Walther et al., 2012; Wang et al., 2016). In line with several other studies, we found reduced FA in the SLF in patients with depression (Ban et al., 2024; Jiang et al., 2017; Murphy & Frodl, 2011; Zeng et al., 2021). A recent neuroimaging study revealed that increased consummatory anhedonia was associated with higher FA values in the SLF in patients with MDD (Coloigner et al., 2019), whilst increased radial diffusivity in regions including the SLF was associated with anticipatory anhedonia (which constitutes an important component of motivational anhedonia) (Yang et al., 2017). Furthermore, reduced FA in right temporal endings of the SLF distinguished patients with anhedonia from those without in bipolar disorder including study participants at familial risk (Diaz et al., 2022). However, in contrast to these findings and reports of other groups investigating associations between white matter microstructure and anhedonia (Bracht et al., 2014, 2015a; Bracht, Mertse, Bracht et al., 2022a, b; Coloigner et al., 2019; Pfarr et al., 2021; Yang et al., 2017), motivational anhedonia did not correlate with FA values in patients with depression, neither in the SLF nor in other regions related to reward processing and action planning (e.g. the reward system). It is possible that our negative finding stems from a mismatch between the regions showing FA reductions and core neural circuits underlying motivational deficits (e.g. prefrontal brain regions and regions of the (dopaminergic) reward system). However, it is of note that previous results are far from conclusive regarding directionality with some groups reporting that lower FA/ reduced tract volume is related to more pronounced anhedonia (Bracht et al., 2014; Bracht, Mertse, Bracht et al., 2022a, b; Yang et al., 2017), whilst others found that lower FA is associated with lower anhedonia or higher hedonic tone (Bracht et al., 2015a; Coloigner et al., 2019; Pfarr et al., 2021). A possible explanation for these discrepancies may be patient heterogeneity regarding clinical presentation and medication status, methodological approaches that differ regarding applied analysis methods (tractography vs. TBSS) and differences regarding investigated aspects of anhedonia due to rating scale heterogeneity (e.g. Snaith Hamilton Pleasure Scale (SHAPS), Fawcett-Clark Pleasure Scale (FCPS), Temporal Experience of Pleasure Scale (TEPS), or items derived from depression rating scales (e.g. BDI) (Treadway & Zald, 2011), which further complicates comparability (Bracht, Linden et al., 2015b).

Finally, our study has some limitations. First, sample size is relatively small. Second, the concept of motivational anhedonia requires better and validated assessment tools (Pizzagalli, 2022; Rizvi et al., 2016; Treadway & Salamone, 2022). However, we elucidate neurobiological aspects of a previously rather neglected aspect of anhedonia by merging components related to motivational anhedonia that were assessed with validated scales (HAMD, CORE). Third, TBSS approaches have limitations because in regions of crossing fibres, it is more difficult to assign clusters to fibre tracts and because TBSS-analysis is restricted to a thinned white matter skeleton. Nevertheless, it is the most established tool to explore whole brain white matter alterations (Smith et al., 2006). Fourth, patients were medicated which may impact motivation, and brain structure and function. This may be particularly true for rs-fMRI-measures (Cheng et al., 2017; Cui et al., 2021), whilst studies investigating the impact of SSRI-treatment on DWI-based measures yielded conflicting results (Davis et al., 2019; Seiger et al., 2021).

## Conclusions

In sum, we found functional alterations of LCOR in bilateral precunei in patients with depression. Our results suggest a (putative) dysfunctional hypoactivation in depression related to motivational anhedonia. There were FA reductions in a cluster localized in the SLF; however, white matter microstructural changes were not related to motivational anhedonia. In sum, our results point to structural and functional alterations in regions associated with complex cognitive functions and goal-directed behaviour, aspects that may well contribute to motivational anhedonia in patients with depression.

## Data Availability

Data are available from the corresponding author by request.

## Code availability

The programming code is available from the corresponding author by request.
